# Randomized controlled trial study of intelligent rehabilitation training system for functional ankle instability

**DOI:** 10.1038/s41598-024-55555-y

**Published:** 2024-02-29

**Authors:** Xiaolong Liu, Mengxiao He, Rongbo Hu, Zhencheng Chen

**Affiliations:** 1 School of Life and Environmental Science, Guilin University of Electronic Technology, Guilin, 541004 Guangxi China; 2 School of Electronic Engineering and Automation, Guilin University of Electronic Technology, Guilin, 541004 Guangxi China; 3 Rehabilitation College, Guilin Life and Health Career Technical College, Guilin, 541001 Guangxi China; 4 School of Physical Education and Health, Guilin University, Guilin, 541006 Guangxi China; 5 Credo Robotics GmbH, Bajuwarenstrasse 47, 94315 Straubing, Germany; 6 Department of System Design Engineering, Keio University, Yokohama, Kanagawa 223-8522 Japan; 7 Guangxi Colleges and Universities Key Laboratory of Biomedical Sensors and Intelligent Instruments, Guilin, 541004 Guangxi China; 8 Guangxi Engineering Technology Research Center of Human Physiological Information Noninvasive Detection, Guilin, 541004 Guangxi China

**Keywords:** Functional ankle instability, Functional recovery, Intelligent rehabilitation training, Randomized Controlled Trial, Rehabilitation effect, Rehabilitation, Biomedical engineering

## Abstract

To investigate the intervention effect of an intelligent rehabilitation training system on patients with functional ankle instability (FAI) and to advance the research to optimise the effect of FAI rehabilitation training. Thirty-four FAI patients who participated in this trial in Guilin City from April 2023 to June 2023 were recruited as research subjects, and all subjects were randomly divided into the control group (n = 17) and the observation group (n = 17). Both groups received the conventional rehabilitation training intervention for 6 weeks, and the observation group received the additional training using the intelligent rehabilitation training system training invented by our team. Visual analogue scale (VAS), ankle active mobility, ankle muscle strength and Y-balance test (YBT) were assessed before and after treatment. Two-way repeated measures ANOVA shows that the interaction effect between time and group of VAS scores was significant (*F* = 35.644, *P* < 0.05). The interaction effect between time and group of plantar flexion mobility was significant (*F* = 23.948, *P* < 0.05), the interaction effect between time and group of dorsiflexion mobility was significant (*F* = 6.570, *P* < 0.05), the interaction effect between time and group of inversion mobility was significant (*F* = 8.360, *P* < 0.05), the interaction effect between time and group of eversion mobility was significant (*F* = 10.113, *P* < 0.05). The interaction effect between time and group of inversion muscle strength was significant (*F* = 18.107, *P* < 0.05). The interaction effect between time and group of YBT scores was significant (*F* = 33.324, *P* < 0.05). The Intelligent Rehabilitation Training System can effectively reduce pain in FAI patients, improve joint range of motion, increase inversion strength, and improve dynamic balance of the affected limb.

## Introduction

Functional ankle instability (FAI) refers to abnormalities in the function of the muscles, ligaments, and nervous system around the ankle joint that result in a loss of normal support and control of the ankle joint during motion and weight bearing, and is a subtype of chronic ankle instability^1^ Freeman first proposed FAI in 1965. The primary characteristic of FAI is the inability to precisely control joint movements, and during movement there is a sensation of soreness in the lower limbs. The prevalence of FAI after lateral ankle sprain is as high as 40%^2^ FAI causes long-term discomfort and limitation of movement for patients and imposes a heavy medical burden on society.

Current treatment modalities for FAI include surgical and non-surgical therapies^3^ rehabilitation training is widely used as a non-invasive treatment for FAI. It shows good therapeutic efficacy^4^ Traditional FAI rehabilitation training mainly includes muscle strength training, neuromuscular control training, balance training, proprioceptive training, etc^5^ Previous studies have concluded that balance rehabilitation training promotes neural adaptation, improves neuromuscular coordination, and strengthens connective tissue^6^ which will have a beneficial effect on the body's perceptual and motor control functions, muscle strength, neuromuscular coordination, and dynamic balance function^7,8^ However, the current balance rehabilitation training still has many problems: shortage of per capita rehabilitation medical resources and heavy workload of therapists, many balance rehabilitation training methods but lack of uniform implementation standards for each training method, the effect of rehabilitation therapy is greatly affected by the differences in human operation of therapists, the privacy between doctors and patients is difficult to protect because there is a lot of physical contact during the treatment process, and it is controversial whether the multimodal rehabilitation training for FAI is effective or not^9–12^.

Our team designed and implemented an intelligent rehabilitation training system (Chinese Invention Patent, Patent No. 2021107165452), and then recruited patients with FAI to conduct a randomized controlled trial to observe the intervention effect of the device on FAI. The study aims to solve the current problems of balance rehabilitation training, promote research on optimizing the effect of balance rehabilitation training, and provide ideas and theoretical references for innovative research on balance rehabilitation training.

## Methods

The study design was a double-blind, randomized, controlled trial, and subjects in each group were unaware of each other’s rehabilitation training programs. Prior to the study, all subjects underwent a physical examination to confirm the absence of certain medical conditions. Subjects signed an informed consent form before participating in the study, and Guilin University approved the study, which met the ethical standards of the Declaration of Helsinki. During rehabilitation training in this study, confounding or effect-modifying factors mainly include the influence of family and work environment on rehabilitation training, patients' cognitive level of rehabilitation, previous rehabilitation experience, etc.

### Research subjects

**Figure 1:**
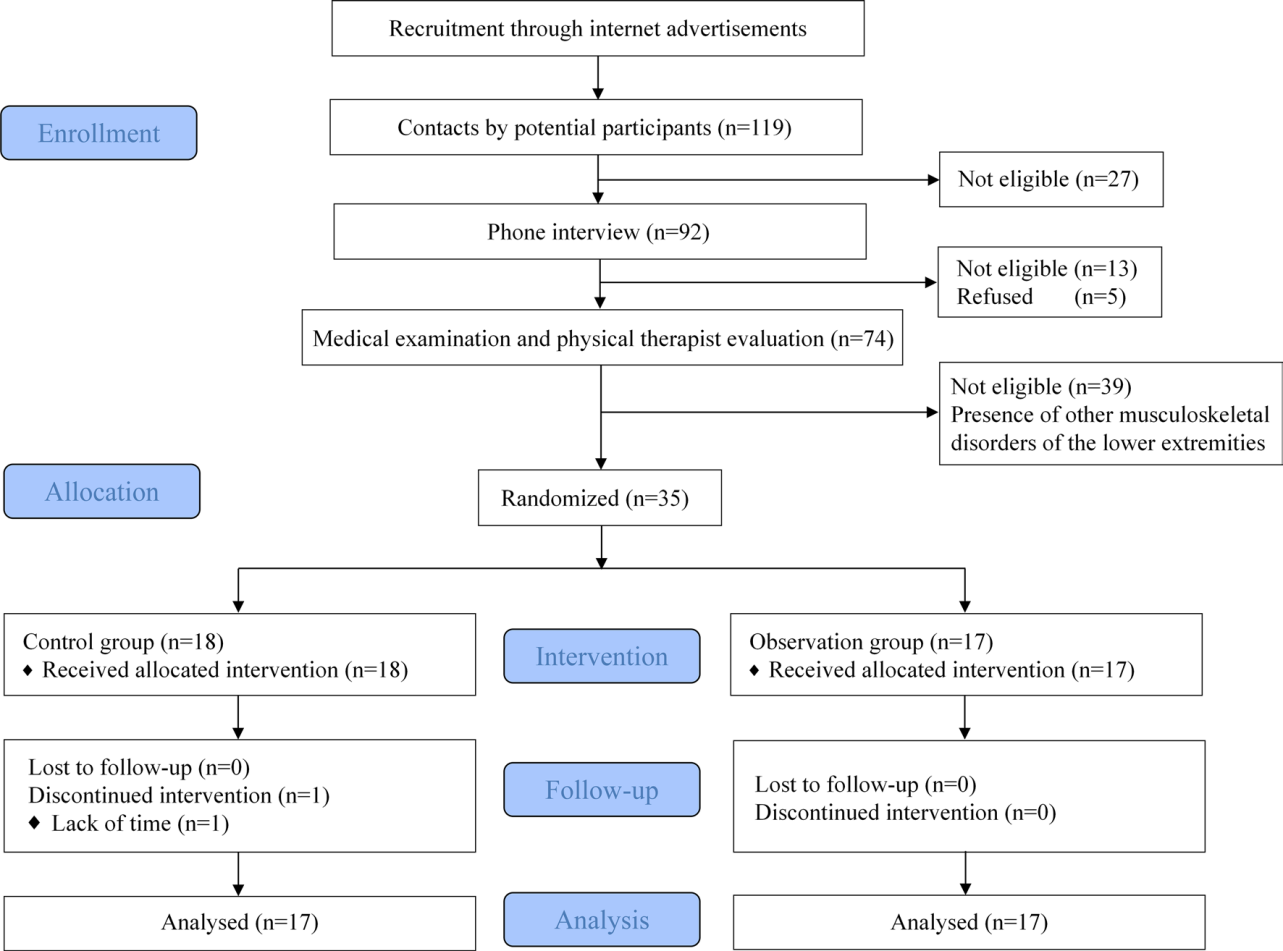
Flow chart of the trial.

We recruited 119 subjects who were willing to participate in this syudy from April 2023 to June 2023 in Guilin City through Internet advertisement. Through the initial screening of basic enrollment information, we excluded 27 enrollees (age exceeded the criteria, personal information was not completely filled out). Subsequently, 92 enrollees were interviewed by telephone and introduced to the study process, and we excluded an additional 13 enrollees who did not meet the study inclusion criteria based on the patient's verbal description of their medical condition, while five participants declined to enroll in the study because they were not interested in our study program. We then called 74 participants who had been interviewed by phone and had them undergo a medical examination and rehabilitation assessment by an experienced physiotherapist at the Sports Rehabilitation Laboratory of Guilin University. Again, 39 participants with other musculoskeletal conditions were excluded from the medical examination and rehabilitation assessment, and the final number of subjects included in the study was 35. We computer-generated a table of random numbers, which was then used to randomly group male and female subjects separately. To keep the grouping hidden, we had an independent person, not directly related to the research team, perform the actual grouping operation. A double-blind method was used to conduct the study, and all information about the Intelligent Rehabilitation Training System and the study design was kept confidential. Randomization was used to divide the 35 subjects into a control group (n = 18) and an observation group (n = 17), with three female subjects in each of the control and observation groups. One male subject in the observation group was excluded due to work commitments that caused him to miss one rehabilitation session. Therefore, a total of 34 subjects were included in the metrics analysis at the end of the six-week study. The mean age of the 34 subjects included in the study was (30.14 ± 7.46) years, and the flow chart of the study is shown in Fig. 1. The study was conducted at the Sports Rehabilitation Laboratory of Guilin University. The study was registered with the China Clinical Trial Registration Center (registration number: ChiCTR2300078652 14/12/2023). The study was reviewed and approved by the Ethics Committee, School of Physical Education and Health, Guilin University (No.GCPEH2023001), and all subjects signed an informed consent form. Inclusion criteria^13^: (1) Age between 18 and 65 years; (2) History of at least 1 significant ankle sprain with significant inflammatory response such as swelling or pain after injury; (3) Most recent sprain occurred at least 3 months prior to study entry; (4) Sensation of loss of ankle control during functional activities at least 2 times in the 6 months prior to study entry; (5) Cumberland Ankle Instability Rating Questionnaire score < 24; (6) Drawer test and ankle inversion stress test did not reveal significant joint laxity; (7) No lower extremity fracture confirmed by x-ray or CT; (8) No rehabilitation therapy received; (9) Clinical examination did not reveal abnormal sensation of ankle depth. Exclusion criteria: (1) History of ankle fracture or surgery; (2) Bilateral ankle instability; (3) Neurological diseases that affect lower extremity muscle strength and balance function; (4) other musculoskeletal diseases that complicate the lower extremity and thus affect life. Cull criteria: (1) Incomplete baseline data, making it impossible to analyze safety and efficacy; (2) Poor adherence to evaluation and treatment; (3) Treatment discontinuation or inability to complete evaluation for any reason. The basic information about the subjects is shown in Table 1.

**Table 1:** Basic information about the subjects.

Group	n	Gender	Age	Height (cm)	Weight (kg)	BMI
Control group	17	M = (14)	32.24 ± 7.46	170.18 ± 7.13	62.47 ± 9.27	21.46 ± 1.90
		F = (3)				
Observation group	17	M = (14)	28.06 ± 7.09	171.35 ± 5.13	64.53 ± 8.29	21.93 ± 2.20
		F = (3)				
*t*-value			1.673	− 0.552	− 0.682	− 0.668
*P*-value			0.104	0.585	0.500	0.509

No statistically significant difference between the two groups.

### Intervention methods

Both groups received conventional rehabilitation training, and the observation group increased their training with the intelligent rehabilitation training system developed independently by our team, for a total intervention period of 6 weeks.

#### Routine rehabilitation training

The routine rehabilitation training consisted of warm-up activities, resistance training exercises, and muscle stretching and relaxation. Training was performed once a day, and each training session was performed on alternate days. The warm-up activities lasted five minutes each and consisted of active joint movements. Resistance training was assisted by elastic bands, and under the guidance of the therapist, resistance training was performed in each direction of ankle plantarflexion, ankle dorsiflexion, ankle inversion, ankle eversion, etc., with 10 times in each direction as one group, making a total of three groups. Muscle stretching and relaxation was performed after each training session, in the form of passive stretching of ankle plantarflexion, ankle dorsiflexion, ankle inversion and ankle eversion muscle groups for 30 s each time, a total of three groups.

#### Intelligent rehabilitation training system training

**Figure 2:**
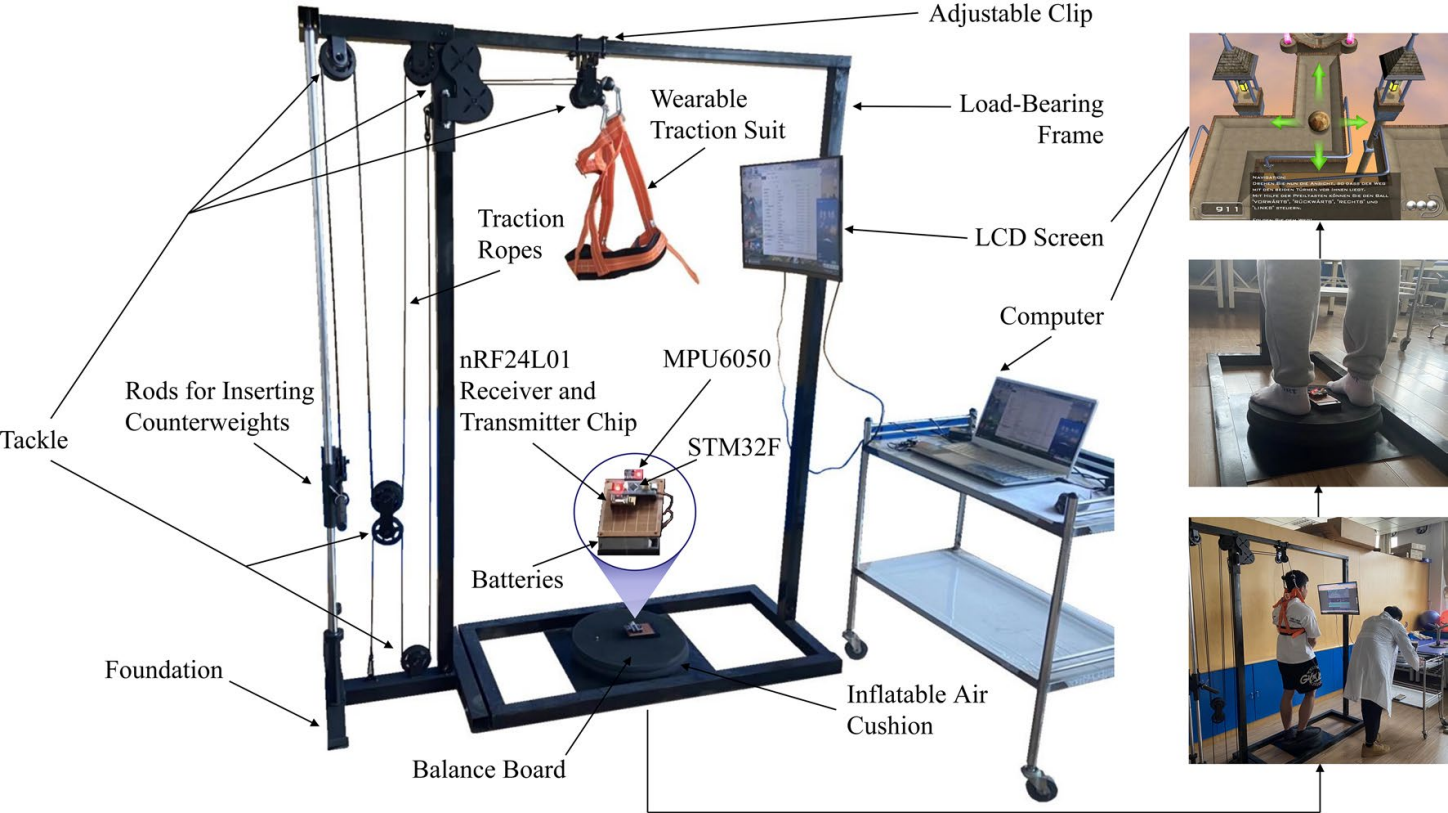
Intelligent rehabilitation training system structure schematic diagram.

**Figure 3:**
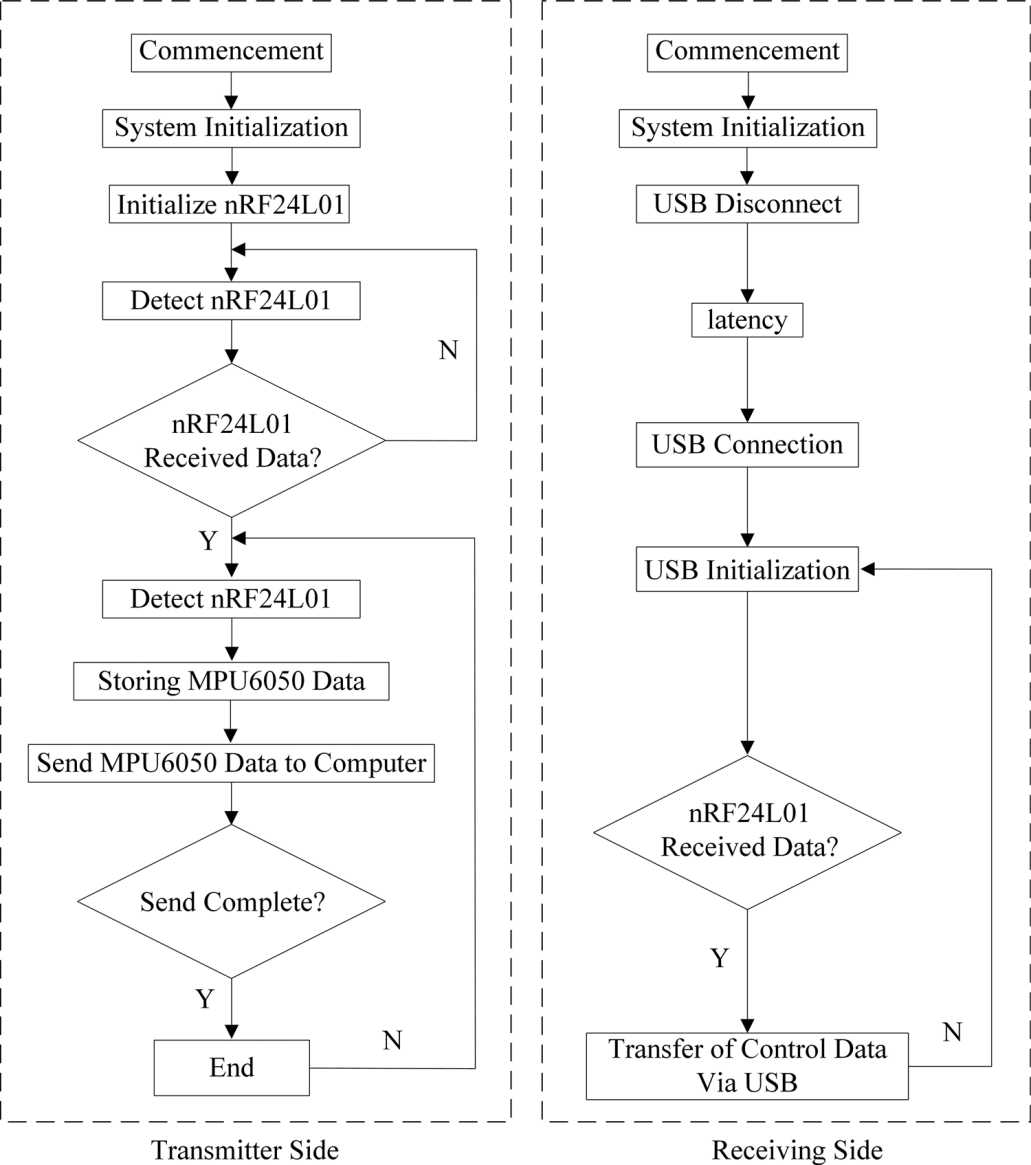
Software control flowchart of the transmitter and receiver side of the system.

The training was performed once a day for 30 min on alternate days. Before the training, the patient can choose whether to wear the traction protective clothing or not, and then the therapist sets the resistance of the intelligent rehabilitation training system according to the patient's rehabilitation needs, followed by setting the type and difficulty of the game. After setting the resistance value, the patient stands on the balance system and adjusts the body balance to control the virtual objects on the LED screen, thus realizing the gamified rehabilitation training. As the treatment progresses, the patient's adaptive ability gradually increases, and the amount of exercise is periodically increased to improve the therapeutic effect. The structure of the intelligent rehabilitation training system mainly includes a computer, LCD screen, pulley traction mechanism, balance board, STM32 chip (manufactured by STMicroelectronics), nRF24L01 receiver-transmitter chip (manufactured by Nordic), batteries, airbags, etc., and the hardware was designed using SolidWorks 3D modeling software. 3D printing technology is used to support the production of prototypes, the overall structure of the system schematic is shown in Fig. 2. The software is written in C language and Keil 4 software, and the software control flow of the transmitter and receiver of the system is shown in Fig. 3.

### Observation indicators

#### Visual analogue scale score (VAS)

Visual Analog Scale (VAS) Score for Pain The VAS was used to assess the patient's level of ankle pain before and after the procedure. A score of zero represents no pain, 10 represents unbearably severe pain, and the higher the score, the worse the pain symptom^14^.

#### Active ankle mobility

The angles of ankle plantarflexion, ankle dorsiflexion, ankle inversion, and ankle eversion were measured separately for all patients before and after the intervention using a joint mobility tape, and the therapist recorded the maximum pain-free range of motion for each maneuver and repeated the measurements for each angle three times, taking the optimal result in the test^15^.

#### Strength of ankle muscles

Ankle strength testing was performed using the American Hoggan MicroFet 2 handheld strength tester (http://www.hoggan.cn/productdetail?product_id=3). Before the test, the subject was instructed to lie supine with the ankle on the test side extended to the bed, and the muscle strengths of ankle plantarflexion, ankle dorsiflexion, ankle inversion, and ankle eversion were measured five times in each direction, and the maximum value was automatically recorded by the system software^16^.

#### Y-balance test score

The distance from the subject's affected medial ankle to the anterior superior iliac spine was measured and recorded as the lower limb length. The patient stood unipedally in the center of the platform, pinched the waist with both hands, the thumb of the foot facing forward against the center line of the anterolateral scale, maintained a unipedal standing posture, the contralateral foot extended as far as possible in all three directions to push the scale, and the values corresponding to the scale were recorded (to the nearest five centimeters), the test was performed three times in each direction and the maximum value was taken. The final score of the YBT was based on the unilateral side, and the average of the scores of the three directions with the length of the lower limb was taken, e.g., if the scores of the three directions and the length of the lower limb are a, b, c, and d, respectively, then the final score is [(a + b + c)/3d] × 100%. If the final score is < 95%, it indicates a high risk of sports injury on that side^17^.

### Statistical analysis

Data were analyzed by SPSS Statistics 26.0 software and expressed as mean ± standard deviation ($\overline{X}$ ± SD). With the experimental procedure (control group vs. observation group) as the between-group variable and the time factor (pre-measurement vs. post-measurement) as the within-group variable, two-way repeated measures analysis of variance was used to test each index, and independent samples t-test was used to test baseline differences in each index, with significance level defined as *P* < 0.05. Data from this study were analyzed by an independent statistician.

## Results

### Comparison of baseline levels

To confirm whether there were differences between the pre-intervention indices of the control group and the observation group, an independent samples t-test was used. Results As shown in Table 2, there was no significant difference between the groups before the intervention.

**Table 2:** Comparison of indices baseline between control group and observation group.

	Index	Control group	Observation group	*t*-value	*P*-value
Pain perception	VAS scores	4.59 ± 1.73	5.41 ± 1.00	− 1.644	0.110
Active ankle mobility (°)	Plantar flexion	49.76 ± 4.48	48.53 ± 4.61	0.792	0.434
	Dorsiflexion	11.71 ± 2.39	11.94 ± 1.14	− 0.366	0.717
	Inversion	24.53 ± 3.66	23.18 ± 5.31	0.865	0.393
	Eversion	18.18 ± 3.70	19.12 ± 3.28	− 0.786	0.438
Muscle strength (Lb)	Plantar flexion	28.88 ± 2.39	29.65 ± 2.52	− 0.907	0.371
	Dorsiflexion	30.59 ± 2.81	31.12 ± 1.90	− 0.644	0.524
	Inversion	20.29 ± 3.14	19.41 ± 3.37	0.790	0.436
	Eversion	16.24 ± 2.39	17.12 ± 2.83	− 0.984	0.333
Dynamic balance	YBT scores	70.49 ± 6.60	68.06 ± 7.65	0.990	0.330

### Comparison of Visual analogue scale score

As shown in Table 3, the interaction effect between time and group of VAS scores was significant (*F* = 35.644, *P* < 0.05). The results of the simple effect analysis show that there was a significant difference in VAS scores between the two groups after the intervention (*F* = 4.571, *P* < 0.05). The VAS scores measured before and after the control group were significantly different (*F* = 53.545 *P* < 0.05), the VAS scores measured before and after the observation group were significantly different (*F* = 248.396 *P* < 0.05).

**Table 3:** Comparison of VAS scores between control and observation group.

		Control group	Observation group	*F*-value	*P*-value	*F*_Time/group/interactive value_	*P*_Time/group/interactive value_
VAS	Pre	4.59 ± 1.73	5.41 ± 1.00	2.872	0.100	266.297/0.018/35.644	0.000/0.894/0.000
	Post	3.06 ± 1.56*	2.12 ± 0.93*^#^	4.571	0.040		
	*F*-value	53.545	248.396				
	*P*-value	0.000	0.000				

*Indicates that there are significant differences in the within-group.

^#^Indicates that there are significant differences between the groups.

### Comparison of active ankle mobility

As shown in Table 4, for active joint mobility, the interaction effect between time and group of plantar flexion mobility was significant (*F* = 23.948, *P* < 0.05), the interaction effect between time and group of dorsiflexion mobility was significant (*F* = 6.570, *P* < 0.05), the interaction effect between time and group of inversion mobility was significant (*F* = 8.360, *P* < 0.05), the interaction effect between time and group of eversion mobility was significant (*F* = 10.113, *P* < 0.05). The results of the simple effect analysis show that there was a significant difference in plantar flexion mobility between the two groups after the intervention (*F* = 6.837, *P* < 0.05), there was a significant difference in dorsiflexion mobility between the two groups after the intervention (*F* = 12.072, *P* < 0.05), there was a significant difference in inversion mobility between the two groups after the intervention (*F* = 7.285, *P* < 0.05), there was a significant difference in eversion mobility between the two groups after the intervention (*F* = 14.711, *P* < 0.05).

**Table 4:** Comparison of active ankle mobility between control and observation group.

		Control group	Observation group	*F*-value	*P*-value	*F*_Time/Group/Interactive value_	*P*_Time/Group/Interactive value_
Plantar flexion(°)	Pre	49.76 ± 4.48	48.53 ± 4.61	0.628	0.434	137.938/0.670/23.948	0.002/0.419/0.000
	Post	53.06 ± 3.93*	56.53 ± 3.81*#	6.837	0.014		
	*F*-value	23.469	138.417				
	*P*-value	0.000	0.000				
Dorsiflexion (°)	Pre	11.71 ± 2.39	11.94 ± 1.14	0.134	0.717	67.068/7.536/6.570	0.000/0.010/0.015
	Post	14.35 ± 1.54*	17.00 ± 2.74*#	12.072	0.001		
	*F*-value	15.828	57.809				
	*P*-value	0.000	0.000				
Inversion (°)	Pre	24.53 ± 3.66	23.18 ± 5.31	0.749	0.393	532.931/0.210/8.360	0.000/0.650/0.007
	Post	37.47 ± 2.62*	39.82 ± 2.46*#	7.285	0.011		
	*F*-value	203.897	337.394				
	*P*-value	0.000	0.000				
Eversion (°)	Pre	18.18 ± 3.70	19.12 ± 3.28	0.617	0.438	182.268/6.027/10.113	0.000/0.020/0.003
	Post	23.24 ± 2.82*	27.29 ± 3.33*#	14.711	0.001		
	*F*-value	53.257	139.125				
	*P*-value	0.000	0.000				

*Indicates that there are significant differences in the within-group.

^#^Indicates that there are significant differences between the groups.

Compared to the control group before and after the intervention, plantar flexion mobility has significant differences (*F* = 23.469 *P* < 0.05), dorsiflexion mobility has significant differences (*F* = 15.828 *P* < 0.05), inversion mobility has significant differences (*F* = 203.897 *P* < 0.05), eversion mobility has significant differences (*F* = 53.257 *P* < 0.05). Compared to the observation group before and after the intervention, plantar flexion mobility has significant differences (*F* = 138.417 *P* < 0.05), dorsiflexion mobility has significant differences (*F* = 57.809 *P* < 0.05), inversion mobility has significant differences (*F* = 337.394 *P* < 0.05), eversion mobility has significant differences (*F* = 139.125 *P* < 0.05).

### Comparison of ankle muscle strength

As shown in Table 5, the interaction effect between time and group of inversion muscle strength was significant (*F* = 18.107, *P* < 0.05). The results of the simple effect analysis show that there was a significant difference in inversion muscle strength between the two groups after the intervention (*F* = 5.051, *P* < 0.05).

**Table 5:** Comparison of ankle muscle strength between control and observation group.

		Control group	Observation group	*F*-value	*P*-value	*F*_Time/Group/Interactive value_	*P*_Time/Group/Interactive value_
Plantar flexion(Lb)	Pre	28.88 ± 2.39	29.65 ± 2.52	0.824	0.371	23.144/0.696/0.146	0.000/0.410/0.705
	Post	32.29 ± 4.33*	33.65 ± 6.64*	0.495	0.487		
	*F*-value	9.808	13.482				
	*P*-value	0.004	0.001				
Dorsiflexion(Lb)	Pre	30.59 ± 2.81	31.12 ± 1.90	0.415	0.524	36.779/0.317/0.000	0.000/0.577/1.000
	Post	33.71 ± 3.57*	34.24 ± 3.85*	0.173	0.680		
	*F*-value	18.390	18.390				
	*P*-value	0.000	0.000				
Inversion (Lb)	Pre	20.29 ± 3.14	19.41 ± 3.37	0.632	0.436	99.323/1.128/18.107	0.000/0.296/0.000
	Post	23.29 ± 3.82*	26.88 ± 5.36*#	5.051	0.032		
	*F*-value	16.307	101.122				
	*P*-value	0.000	0.000				
Eversion (Lb)	Pre	16.24 ± 2.39	17.12 ± 2.83	0.968	0.333	23.642/0.975/0.243	0.000/0.331/0.625
	Post	18.06 ± 2.99*	19.35 ± 4.96*	0.849	0.364		
	*F*-value	9.544	14.341				
	*P*-value	0.004	0.001				

*Indicates that there are significant differences in the within-group.

^#^Indicates that there are significant differences between the groups.

Compared to the control group before and after the intervention, plantar flexion strength has significant differences (*F* = 9.808 *P* < 0.05), dorsiflexion strength has significant differences (*F* = 18.390 *P* < 0.05), inversion strength has significant differences (*F* = 16.307 *P* < 0.05), eversion strength has significant differences (*F* = 9.544 *P* < 0.05). Compared to the observation group before and after the intervention, plantar flexion strength has significant differences (*F* = 13.482 *P* < 0.05), dorsiflexion strength has significant differences (*F* = 18.390 *P* < 0.05), inversion strength has significant differences (*F* = 101.122 *P* < 0.05), eversion strength has significant differences (*F* = 14.341 *P* < 0.05).

### Comparison of Y-balanced test scores

As shown in Table 6, the interaction effect between time and group of YBT scores was significant (*F* = 33.324, *P* < 0.05). The results of the simple effect analysis show that there was a significant difference in YBT scores between the two groups after the intervention (*F* = 6.254, *P* < 0.05). The YBT scores measured before and after the control group were significantly different (*F* = 152.707 *P* < 0.05, the YBT scores measured before and after the observation group were significantly different (*F* = 421.126 *P* < 0.05).

**Table 6:** Comparison of Y-balanced test scores between control and observation group.

		Control group	Observation group	*F*-value	*P*-value	*F*_Time/Group/Interactive value_	*P*_Time/Group/Interactive value_
YBT	Pre	70.49 ± 6.60	68.06 ± 7.65	0.980	0.330	540.509/0.686/33.324	0.000/0.414/0.000
	Post	83.80 ± 8.24*	90.17 ± 6.51*^#^	6.254	0.018		
	*F*-value	152.707	421.126				
	*P*-value	0.000	0.000				

*Indicates that there are significant differences in the within-group.

^#^Indicates that there are significant differences between the groups.

## Discussion

This section will systematically analyze the reasons for the changes in each indicator in the control and observation groups.

### Changes in pain perception

At baseline, there was no difference in VAS scores between the control and observation groups. After 6 weeks of intervention, the interaction effect between time and group of VAS scores was significant, indicating that the intelligent rehabilitation training system can effectively reduce pain in FAI patients. Pain perception after acute ankle sprains decreases significantly in the first 2 weeks after injury and the rate of pain reduction decreases thereafter, with approximately 33% of patients still reporting pain at one year^18^. Previous studies have shown that the long-term cause of ankle pain in FAI patients is sensory hypersensitivity, a sensory nerve abnormality that can be seen arthroscopically as chronic inflammation of the synovial membrane that causes causing pain when locally stimulated, and that prolonged inflammatory stimulation can lead to peripheral sensitisation of the nerves or alter the central nervous system’s perception of pain to appear as central sensitisation^19^. On the other hand, ankle injuries cause damage to the proprioceptors in the soft tissues, resulting in decreased body stability, proprioception and muscle coordination, which increases the likelihood of re-injury to the ankle and the vicious cycle of injury-decreased proprioception-re-injury-pain^20^. Studies have shown that balance training is effective in reducing pain after ankle sprains. Lazaros et al. found that balance can significantly increase dorsiflexion range of motion in patients with ankle sprains and reduce patients' subjective levels of pain^21^. There is also strong evidence that balance and coordination training reduces the risk of ankle sprains^22,23^. Progressive exercise is the targeted manipulation of stress to promote cellular health and tissue repair and healing, the intelligent rehabilitation training system has a balance training function, the reduction of subjective pain in the ankle joint may be due to the regular contraction and relaxation of the target muscle groups during training, the soft tissues adhering to the periphery of the joint are pulled, resulting in vasodilation, the organisation of the local blood circulation function has been improved, which promotes the transport of acid metabolites and inflammatory substances^24^. In addition, balance rehabilitation training can stimulate the peripheral proprioceptors of the ankle, feed back information to the central system and integrate it into the effector so that the ankle can produce timely and correct movement control, modify protective reflexes and increase ankle stability, which can reduce the likelihood of ankle pain^25^. Questions about balance training focus on dose control and type of training, and there are still no conclusions about how often patients should be rehabilitated and what forms of exercise should be used. Although there is no clear answer in the literature as to which exercises are most beneficial, it is a fact that the longer the patient participates in progressive balance and coordination training, the greater the response. To maximise the benefits of exercise, we recommend that the exercise prescription is individualised to the patient and disease process.

### Changes in active ankle mobility

At baseline, there was no difference in ankle mobility between the control and observation groups. After 6 weeks of intervention, the interaction effect between time and group of plantar flexion mobility, dorsiflexion mobility, inversion mobility, and eversion mobility was significant, indicating that the intelligent rehabilitation training system can effectively improve ankle range of motion. Patients with FAI generally have deficits in proprioception strength perception and kinaesthesia, and the majority of patients with FAI have active joint position sensory deficits and kinematic deficits of ankle inversion and ankle eversion^26^. On the other hand, after an ankle inversion injury, the soft tissues produce an inflammatory response that leads to joint adhesion, resulting in a decrease in joint mobility, and at the same time the patient will protectively reduce the activity of the injured joint to reduce the subjective pain, and prolonged braking will lead to a further decrease in joint mobility^27^. In addition, reduced joint range of motion has been linked to deficits in dynamic postural control deficits, reduced proprioception, ligamentous laxity, and other multifactorial factors^28^. Negahban et al. found that adding balance training to ankle rehabilitation was more conducive to improve ankle functions^29^. Sasaki et al. found that core stability training can increase the range of trunk flexion and improve the biomechanical properties of the lower limbs and trunk^30^. The intelligent rehabilitation training system gradually explores the stability limits of FAI patients through progressive and ever-changing interesting tasks. By controlling the virtual objects to complete swinging and rotating movements, this exciting balance training improves the body’s neural control function. At the same time, because the intelligent rehabilitation training system has a greater range of motion due to the existence of the unstable plane, it can make the patient mobilise more muscle groups to participate in rehabilitation training, which will promote the return of joint range of motion to normal levels. Because the human body is in a standing position during exercise, too much joint movement also increases the risk of injury. The follow-up study can therefore develop a kind of algorithm. After entering basic information such as height, weight and medical history before training, the computer can automatically adjust the range of motion of the intelligent rehabilitation board.

### Changes in ankle muscle strength

At baseline, there was no difference in ankle muscle strength between the control and observation groups. After 6 weeks of intervention, the interaction effect between time and group of inversion muscle strength was significant, indicating that the intelligent rehabilitation training system can effectively improve ankle inversion strength. Several studies show that FAI patients have deficits in ankle inversion and eversion strength^31^. Muscle weakness as a pathogenetic mechanism and symptom of FAI has been present throughout the development of FAI and previous studies have confirmed its multifactorial association with soft tissue damage, poor muscle coordination, abnormal neural control and pain inhibition. Alizamani et al. observed that core stability training can effectively increase plantar flexion, dorsiflexion, inversion and eversion muscle strength in athletes with FAI^32^ Wang et al. found that 6 weeks of isokinetic strength training and Thera-Band resistance training can effectively increase medial and lateral piriformis muscle strength in FAI patients^33^. Increased muscle-nerve control was once thought to be the main reason for increased muscle strength^34^. After an ankle injury, due to the interference of chronic pain and other factors, the ability of the nervous system to control the muscles around the ankle joint will reduced^35^. In this study, the improvement in muscle strength in FAI patients may be related to the recovery of neuromuscular function. Balance training requires patients to constantly adjust their body movements to cope with the swaying, and with prolonged repetition patients gain experience from previous training and then use this experience to cope with subsequent tasks. This learning effect means that more muscles of FAI patients are pre-activated before the strength test^36^. Conventional rehabilitation training involves resistance training with elastic bands in all directions, which could be effective in increasing muscle strength. In addition, the improvement in ankle varus strength with the intelligent rehabilitation training system may be related to high-frequency varus training.

### Changes in dynamic balance

At baseline, there was no difference in YBT scores between the control and observation groups. After 6 weeks of intervention, the interaction effect between time and group of YBT scores was significant, indicating that the intelligent rehabilitation training system can effectively improve dynamic balance in FAI patients. FAI has deficits in muscle strength, proprioception and neuromuscular control and is therefore at higher risk of somatic sports injuries^37^. Ankle instability occurs during dynamics and YBT identifies dynamic postural control deficits in FAI patients^38^. Previous studies have shown that patients with FAI have reduced spinal reflex regulation and corticospinal excitability of the soleus muscle compared to healthy individuals, which can lead to reduced balance performance. Chung et al. found that balance training can increase soleus reflex regulation and corticospinal excitability in FAI patients^39^. Study shows that balance training leads to positive neural adaptations and improved balance in FAI patients. Hu et al. believe that balance training can induce adaptations in the spinal cord and all sensory systems, and leading sensory reorganisation^40^ Diaz et al. found that balance training can effectively improve dynamic balance and self-reported instability in FAI patients^41^ The effect of the intelligent rehabilitation training system on improving dynamic balance may be due to the fact that patients remain in a standing position for a long time during training, allowing them to adapt to the YBT measurement method in advance. On the other hand, the unstable plane of the intelligent rehabilitation training system can effectively increase the proprioceptive input and effectively improve the ability to resist the interference of the external environment on the centre of gravity and the dynamic control of posture.

## Limitations

Although this study has produced some important findings, there are inevitably some limitations. First, the sample size included in this study was small and the age range of the participants was small, which may have partially influenced the results of the study. Future studies may consider increasing the sample size and demographic diversity to explore in depth the intervention effects of the intelligent rehabilitation training system on different populations and patients of different ages, thus improving the broad applicability of the study. In terms of data collection and measurement, the pain measurement tool VAS score has shortcomings such as high subjectivity, insufficiently refined pain grading and poor repeatability, so we cannot completely exclude the possibility of measurement error. Secondly, due to time constraints, this study only lasted for 6 weeks, and the time span can be extended in the future to observe and analyze the development and changes of the indicators more comprehensively. In addition, this study focused on functional recovery but not on structural changes of the body, we will continue to explore the role of intelligent rehabilitation training system on the ankle joint, and continue to optimise the structure and function of the equipment to promote the iterative improvement of the equipment.

## Conclusion

The Intelligent Rehabilitation Training System can effectively reduce pain in patients with FAI, improve active range of motion of the joint, increase ankle inversion strength and improve dynamic balance of the affected limb.

## Data Availability

The datasets used and/or analysed in the current study are not publicly available due to confidentiality issues, but are available from the corresponding author upon reasonable request.
